# Breast asymmetry and predisposition to breast cancer

**DOI:** 10.1186/bcr1388

**Published:** 2006-03-20

**Authors:** Diane Scutt, Gillian A Lancaster, John T Manning

**Affiliations:** 1 Division of Medical Imaging, University of Liverpool, Liverpool L69 3BX, UK; 2 Centre for Medical Statistics and Health Evaluation, University of Liverpool, Liverpool, UK; 3 Department of Psychology, University of Central Lancashire, UK Preston, Lancashire

## Abstract

**Introduction:**

It has been shown in our previous work that breast asymmetry is related to several of the known risk factors for breast cancer, and that patients with diagnosed breast cancer have more breast volume asymmetry, as measured from mammograms, than age-matched healthy women.

**Methods:**

In the present study, we compared the breast asymmetry of women who were free of breast disease at time of mammography, but who had subsequently developed breast cancer, with that of age-matched healthy controls who had remained disease-free to time of the present study. The study group consisted of 252 asymptomatic women who had normal mammography, but went on to develop breast cancer. The control group were 252 age-matched healthy controls whose mammograms were also normal and who remained free of cancer during the study period. Breast volume was calculated from the cranio-caudal mammograms for each group, and the relationships between asymmetry, established risk factors and the presence or absence of breast cancer were explored.

**Results:**

The group who went on to develop breast cancer had higher breast asymmetry than controls (absolute asymmetry odds ratio 1.50 per 100 ml, confidence interval (CI) 1.10, 2.04; relative asymmetry 1.09, CI 1.01, 1.18), increased incidence of family history of breast cancer, lower age at menarche, later menopause, later first pregnancies and a higher frequency of high risk breast parenchyma types. Conditional logistic regression analysis showed that breast asymmetry, height, family history of breast cancer, age at menarche, parenchyma type and menopausal status were significant independent predictors of breast cancer. When age at menopause was included in the model for the subgroup of post-menopausal women, absolute breast fluctuating asymmetry (FA) and relative breast FA remained significant effects.

**Conclusion:**

Breast asymmetry was greater in healthy women who later developed breast cancer than in women who did not.

## Introduction

Humans, in common with most other vertebrates, show bilateral symmetry in paired morphological traits such as ear size, digit length and breast volume. Perfect symmetry may be disturbed by a number of intrinsic and extrinsic factors, including the secretion of hormones such as oestrogen [1,2]. The small, random deviations from perfect symmetry that result from such factors are termed fluctuating asymmetry (FA). 'Fluctuating' refers to a pattern of bilateral variation where variation on the right and left sides is both random and independent. It tends to be small (around 1% of trait size or less). These random departures from bilateral symmetry provide a surprisingly convenient measure of developmental precision: the more precisely each side develops the greater the symmetry. FA is a measure of developmental stability, and is one of many issues at the interface between biology and medicine that offer valuable information at the whole organism level. Such comprehensive information is a concept familiar to, and frequently used by, biologists, but is often overlooked in medicine [3,4]. Highly symmetrical human and non-human animals are preferred as mates in comparison to asymmetrical individuals [5-9]. If individuals that experience stress during ontogeny are less preferred as potential mates, then presumably mechanisms that reduce stressful developmental events would be favoured [10].

FA in such traits as ear size and digit length is related to health measures including body mass index (BMI) in young women and men [11]. However, it is in sexually selected traits, such as breasts, that the highest values of FA may be found [12-14]. Sexually selected traits are more liable to be disrupted during development because they often show rapid growth rates, are generally elaborate in design and are highly susceptible to mutation that results from rapid cellular proliferation and the action of sex steroids [15-17]. Breasts develop rapidly just prior to and during puberty and the importance of estrogen in the development, growth and carcinogenesis of the mammary gland is well established [18]. The role of local estrogen production in breast cancer is now more apparent [19-22]. Symmetrical breast development may well be an indicator of an individual's ability to tolerate 'disruptive' hormonal variation whilst maintaining developmental stability. Møller and colleagues [23] found that large breasts had more FA than small breasts, breast FA was higher in nulliparous women, and that breast FA was a predictor of fecundity.

Breast cancer is the most common malignancy among women in Western society, with incidence rates continuing their upward trend, increasing by 70% cent since 1971, and by 15% in the 10 years to 2000 in England. It is the most common cause of cancer death in women [24]. There are large between-individual differences in size and asymmetry of breasts and this could be indicative of differences in developmental stability, and possibly disease predisposition.

Breast volume FA, as measured from mammograms, is related to several of the known risk factors for breast cancer [1,2], and patients with diagnosed breast cancer have higher breast volume FA measured from mammography than age-matched healthy women [25]. It would be an important advance if additional variations in the normal mammogram, that is breast asymmetry, could be used to help predict the possibility of developing breast cancer, particularly in high risk individuals. The specific aim of the present study was to establish whether breast FA, as measured from mammograms, was greater in healthy women who later went on to develop breast cancer, compared to age-matched women who remained disease-free.

## Materials and methods

This was a retrospective study on women who had mammography during the period 1979 to 1986. The original study collected detailed breast cancer risk factor data from a total of 12,942 women self-referred to the Liverpool Breast Screening Unit between 1979 and 1986, the aim being to establish whether mammographic parenchymal patterns were associated with hypothesised risk factors for breast cancer [26,27]. The women in the study were asymptomatic of serious breast disease at point of entry, as women symptomatic of breast cancer on referral were filtered out of the study and referred to a surgical clinic. Two view mammography was undertaken on each woman at a single centre. The null hypothesis for the present study was that there would be no difference in breast volume asymmetry between those women who developed breast cancer and those who did not. A subset of women who had subsequently gone on to develop breast cancer during the intervening years to 2002 were identified by matching women in the cohort with breast cancer records from the Merseyside and Cheshire Cancer Registry, which was the single registry covering the original study recruitment area. This resulted in 302 confirmed matched records for breast cancer registration. All recorded cancers are proven by histology, clinical findings or imaging before entry to cancer registry databases. Cranio-caudal mammograms were available for 262 cases, and of these, three women had very large breasts that were not included in their entirety on the films, and seven women had undergone mastectomy prior to the original mammogram. These 10 were therefore excluded, leaving 252 subjects. This group comprised 144 peri- or post-menopausal women, 99 pre-menopausal women and 9 for whom the menopausal status was not recorded.

Breast volume (ml) was calculated from cranio-caudal mammograms of the 252 patients who had subsequently developed breast cancer, and 252 age-matched controls, as breast FA increases with age [2]. The controls were from the same risk factor study, none of whom had developed breast cancer to date and were age-matched within 37 one-year age groups ranging from 33 to 70 years at date of mammography. Where possible, an equal number of controls were selected for the number of cases within each age group, resulting in between 1 and 21 controls per age group stratum. The controls were randomly selected from an alphabetical list of potential women for that stratum. For two age strata no controls were found, resulting in four cases being included in the age stratum for the previous year. The control group was composed of 163 peri- or post-menopausal women, 76 pre-menopausal subjects, and 13 subjects for whom menopausal status was not recorded. Mean age at mammography for the control group was 48.60 years (standard deviation (SD) 6.95) with a range of 33 to 70 years, and for the cancer group was 48.77 (SD 7.25) with a range of 33 to 70 years. Measurements of breast width (maximum width of breast tissue along proximal edge of cranio-caudal film) and breast height (maximum perpendicular distance from the distal edge of the breast to the proximal edge of the film) were performed directly from the films by two reviewers, blinded to the case-control status. Risk factor data had been collected for each subject by questionnaire, administered by a breast nurse counsellor at the time of mammography. These included family history of breast cancer, age at menarche, number of pregnancies, age at first pregnancy, duration of breast feeding, weight, height and breast parenchyma type as described by Wolfe in 1976 [28].

The method employed for breast volume calculation from the mammograms was that used by Katariya and colleagues [29] and Hoe and colleagues [30], which is highly reproducible [31]. The formula used for calculating the volume was that for the calculation of the volume of a cone:

π r^2^h

where r was half the breast width and h the breast height. FA of breast volume was calculated by subtracting the right volume measure from that of the left (L - R).

FAs from linear measurements of mammograms have been shown to correlate significantly with FAs calculated from direct measurements of the breasts [1]. Subjects with larger left than right breasts have +FAs, and those with larger right than left breasts have -FAs. If asymmetries are 'ideal' FAs, the signed asymmetries are normally distributed around a mean of zero [32]. Data analysis was on unsigned (absolute) values (|L - R|) of breast volume FA. Breast FA is strongly related to breast volume [2] so we also calculated relative FA. Relative FA is defined as a percentage of average breast size and was calculated as:



Mammograms had been rated for increasing density of breast parenchyma tissue by a single reporting radiologist using the classification of Wolfe [28] where: N1 = lowest risk, parenchyma primarily fat; P1 = low risk, parenchyma chiefly fat with prominent ducts up to a quarter of the breast; P2 = high risk showing severe involvement of the breast with prominent duct pattern occupying more than a quarter of the breast; DY = highest risk showing severe involvement with dysplasia, often obscuring an underlying prominent duct pattern. Two readings were carried out on separate occasions, without the knowledge of the previous code assigned. The concordance rate between the readings was 97.8%. When the code differed on two occasions the films were subjected to further scrutiny before a final coding was allocated.

The data were analysed using the SPSS v.13 and Stata v.8.2 statistical packages (SPSS Inc., Chicago, Illinois, USA; StataCorp. LP, Texas, USA). Using two conditional logistic regression models (for absolute breast FA and relative breast FA) the crude and adjusted relative odds of breast cancer were estimated for each breast FA variable and other known potential risk factors and confounders, together with 95% confidence intervals (95% CI). For the purposes of this analysis, because a 1 ml change in breast volume FA is very small, the results for breast volume FA are presented as the change in relative odds for a 100 ml change in breast volume FA. In addition, given the strong effect of age at menopause on breast cancer risk, this was controlled for in additional models containing only post-menopausal women. The effect of age at first pregnancy was modelled using another set of conditional logistic regression models for the subset of women who had ever been pregnant, and again age at menopause was controlled for in an additional analysis including only post-menopausal, ever pregnant women. A case-control stratum was excluded from the analysis if the information for either the cases or controls was not known for the variable in question. The variables were entered together into the multivariable regressions, and then factors that did not contribute significantly after consideration of other potential cofactors were removed from the model only if they were found not to influence the FA associations. The models containing all risk factors are presented and only minor changes to the parameter estimates in the main findings resulted if insignificant terms were removed.

## Results

The mean age at diagnosis of breast cancer was 55.20 (SD 7.84; range 37 to 77 years). The mean interval between mammography and presentation of the tumour was 6.44 years (SD 3.90; range 0 to 15 years). There were 120 right sided tumours, 119 left sided tumours, 6 cases of bilateral disease and 7 where the side was not recorded.

Repeatabilities (intra-class correlation coefficients, *r*_1_) of FAs calculated from mammograms were calculated on a random sub-set of 20 mammograms from the study and showed highly significant *r*_1 _values (repeated measures ANOVA, 20 sets of mammograms, FA breast width *r*_1 _0.98, *p* = 0.0001; FA breast height *r*_1 _0.86, *p* = 0.0001). Ideal FA shows a normal distribution with a parametric mean at zero [32]. Distribution of the signed FAs was tested for normality (skewness *g*_1 _and kurtosis *g*_2_) [33] and deviation from a mean of zero (one sample t-test with the mean set at zero). There was a tendency for left breasts to be slightly larger than right in the sample and the signed asymmetries showed ideal FA [32,33]. The mean ratio of left:right breast size in the whole sample was 1.04 (SD 0.18), in the cancer group 1.05 (SD 0.21), and in controls 1.03 (SD 0.16).

The characteristics of the cases and controls are detailed in Table 1. Breast volume asymmetry was higher in the group that developed breast cancer than those who did not (cancer group breast volume asymmetry median = 63.17 ml (inter-quartile range (IQR) 88.54); control group median = 52.99 ml (IQR 68.38)). As breast FA is strongly related to breast volume [2], FA was also corrected for breast size and showed similar differences between the cancer and control groups, with the former having higher relative breast volume FA (cancer group median = 2.7% (IQR 3.6%); control group median = 2.5% (IQR 2.9%)).

The difference in breast asymmetry between left- and right-sided tumour groups was not significant. For those women who developed left-sided tumours, the right breast was larger at the time of mammography in 57 cases, and the left larger in 57 cases. For those who developed right sided tumours, there were 53 larger right breasts and 64 larger left. The mean BMI was similar for the cancer group and the controls, as was mean breast volume.

Many of the risk factors for breast cancer are interrelated, so the cancer group and controls were entered into a conditional logistic regression analysis, firstly with independent variables absolute breast FA, family history, mean breast volume, BMI, height, age at menarche, number of pregnancies, menopausal status and parenchyma type and secondly with relative breast FA in place of absolute FA. Right breast parenchyma type was used in these analyses as there was little difference between the left and right parenchyma types. The regression results are shown in Table 2.

Table 2 indicates that breast asymmetry (whether absolute or relative), height, family history of breast cancer, age at menarche, parenchyma type and menopausal status were significant independent predictors of breast cancer. The relative odds of breast cancer increased by 1.50 for a one 100 ml increase in absolute breast FA and by 1.09 for a 1% increase in relative breast FA after adjusting for the other potential risk factors. The exclusion of the pre-menopausal and menopausal status not known groups in a sensitivity analysis did not change the findings. When age at menopause was included in the model for the subgroup of post-menopausal women, absolute breast FA and relative breast FA remained significant effects with age at menopause and family history. BMI, height and number of pregnancies were insignificant effects, and mean breast volume and parenchyma type were of borderline significance (results not shown). There was no significant relationship between breast asymmetry and time interval from negative mammogram to diagnosis in the breast cancer group as a whole, but in the subset of women who had died of breast cancer to time of study (*n* = 51), breast asymmetry had a significant inverse relationship with time from mammogram to diagnosis (r = -0.376, *p* = 0.007).

## Discussion

These findings are the first evidence that breast asymmetry is higher in healthy women who are free of breast disease but subsequently go on to develop breast cancer than in women who remain disease-free in the same period. Both absolute and relative breast volume asymmetries were higher in the group that went on to develop cancer than in the control group. This is consistent with our previous findings that women with already diagnosed breast cancer had higher breast FAs than healthy women [25]. The results of our two previous studies suggested that breast asymmetry may be of additional value in the identification of women with an increased risk of developing breast cancer, as it is easily measured from mammograms at the time of reporting [1,25]. In the light of the findings of the present study, the differences in FA between the cancer and control groups found in the two previous studies were probably conservative.

Of the other breast cancer risk factors examined, family history, age at menarche and parenchyma type showed significant differences between the two groups. The cancer group had lower menarchal age than the controls, and a higher frequency (75% of the cancer group compared to 64% of controls) of high risk parenchymal patterns (P2 and DY). The probability that a woman will develop breast cancer is dependent in part on the type and duration of oestrogen exposure, which in turn relates to many of the known risk factors for the disease. Many studies have sought to establish the importance of parenchymal patterns as a predictor of breast cancer. The majority have shown an increased risk associated with increased breast density patterns [28,34-39]. There is more recent compelling evidence that there is a greatly increased occurrence of breast cancers in mammographically dense tissue [40] and that this has high heritability [41].

FA is a measure of one component of fitness, namely, the developmental stability of an individual. Stable development is difficult to maintain if an individual has harmful mutations, or if normally bilaterally symmetrical structures undergo rapid periods of growth [42,43]. The homeostatic mechanisms maintaining symmetry tend to break down during such periods. Growth rate, particularly during puberty, is positively related to FA [44], and breast FA is substantially higher than the FA of such traits as the size of fingers, ears, wrists and other non-sexually selected traits [1,23,45]. How well an individual can maintain a developmental target of symmetry, despite perturbations from influencing factors such as oestrogen may be a reflection of their phenotypic quality.

## Conclusion

In this study, we found that breast asymmetry was greater in the healthy women who subsequently developed breast cancer than those who remained disease free. Asymmetrical breasts could prove to be reliable indicators of future breast disease in women and this factor should be considered in a woman's risk profile.

## Abbreviations

BMI = body mass index; FA = fluctuating asymmetry; IQR = inter-quartile range.

## Competing interests

The authors declare that they have no competing interests.

## Authors' contributions

DS carried out the data collection and drafted the manuscript. DS and JTM both participated in the design of the study and undertook the asymmetry measurements. DS, JTM and GL performed the statistical analysis. JTM and GL helped to draft and edit the manuscript. All authors read and approved the final manuscript.

## Figures and Tables

**Table 1:** Characteristics of women with breast cancer and their matched controls

Variable^a^	Cases of cancer (*n* = 252)	Controls (*n* = 252)
Hypothesised risk factors		
Absolute breast volume FA (ml; median (IQR))	63.17 (88.54)	52.99 (68.38)
Relative breast volume FA (%; median (IQR))	2.70 (3.63)	2.49 (2.91)
		
Other potential risk factors/confounders		
BMI (*n* = 503; mean (SD))	23.8 (3.11)	23.9 (3.22)
Height (cm; mean (SD))	161.7 (6.10)	159.7 (5.88)
Age at menarche (years; *n* = 503; mean (SD))	13.0 (1.61)	13.6 (1.68)
Age at 1st pregnancy (years; *n* = 400; mean (SD))	25.4 (4.19)	23.9 (4.31)
Number of pregnancies (*n* = 503; median (IQR))	2 (2)	2 (2)
Family history	68 (27%)	32 (13%)
Mean breast volume (ml; median (IQR))	569 (388)	582 (370)
Parenchyma type: right breast		
N1	21 (8%)	54 (21%)
P1	42 (17%)	33 (13%)
P2	65 (26%)	40 (16%)
DY	124 (49%)	125 (50%)
Parenchyma type: left breast		
N1	21 (8%)	55 (22%)
P1	42 (17%)	33 (13%)
P2	65 (26%)	43 (17%)
DY	124 (49%)	121 (48%)
Menopausal status (*n* = 482)		
Pre	99 (41%)	76 (32%)
Peri	44 (18%)	49 (20%)
Post	100 (41%)	114 (48%)
Age at menopause (years; *n* = 212; median (IQR))	50 (4)	47 (6)

Values are numbers (percentages) unless stated otherwise. ^a^Incomplete total is indicated in brackets where there are missing data. Absolute breast volume fluctuating asymmetry (FA) = unsigned values (|L - R|) of breast volume. Relative breast volume FA = 100 × |L - R| breast volume/[|L + R| breast volume] 0.5. BMI, body mass index; IQR, inter-quartile range; SD, standard deviation.

**Table 2:** Conditional logistic regression analyses with presence or absence of cancer as the dependent variable, for both absolute and relative breast fluctuating asymmetry (*n* = 504)

Risk factor	Crude relative odds	(95% CI)	*p*-value	Adjusted relative odds^a^	(95% CI)	*p*-value	Adjusted relative odds^b^	(95% CI)	*p*-value
Absolute breast FA (100 ml)	1.29	(1.01, 1.64)	0.039	1.50	(1.10, 2.04)	0.010	-	-	-
Relative breast FA (%)	1.07	(0.99, 1.14)	0.070	-	-	-	1.09	(1.01, 1.18)	0.032
									
BMI	1.00	(0.95, 1.06)	0.98	1.00	(0.91, 1.09)	0.97	1.00	(0.92, 1.09)	0.98
Height (cm)	1.06	(1.03, 1.09)	<0.001	1.06	(1.02, 1.09)	0.001	1.05	(1.02, 1.09)	0.002
Age at menarche (years)	0.81	(0.72, 0.90)	<0.001	0.78	(0.69, 0.89)	<0.001	0.78	(0.69, 0.89)	<0.001
Number of pregnancies	0.94	(0.84, 1.04)	0.23	1.04	(0.92, 1.18)	0.49	1.04	(0.92, 1.18)	0.52
Family history (no. of relatives)	2.00	(1.37, 2.94)	<0.001	2.08	(1.35, 3.20)	0.001	2.10	(1.36, 3.23)	0.001
Mean breast volume (ml)	1.00	(0.999, 1.001)	0.82	1.00	(0.999, 1.000)	0.33	1.00	(0.999, 1.001)	0.94
Parenchyma type: right breast									
N1	1.0	-	-	1.0	-	-	1.0	-	-
P1	3.25	(1.62, 6.52)	0.001	3.23	(1.51, 6.91)	0.003	3.30	(1.54, 7.05)	0.002
P2	4.27	(2.23, 8.17)	<0.001	3.61	(1.73, 7.53)	0.001	3.75	(1.80, 7.82)	<0.001
DY	2.69	(1.51, 4.79)	0.001	2.94	(1.48, 5.84)	0.002	3.02	(1.52, 5.99)	0.002
Menopausal status									
Pre	1.0	-	-	1.0	-	-	1.0	-	-
Peri	0.49	(0.26, 0.91)	0.023	0.45	(0.23, 0.87)	0.017	0.46	(0.24, 0.89)	0.022
Post	0.29	(0.14, 0.61)	0.001	0.37	(0.16, 0.83)	0.017	0.38	(0.17, 0.85)	0.018
Age at menopause^c ^(years)	1.16	(1.06, 1.27)	0.002	1.18	(1.06, 1.31)	0.002	1.17	(1.06, 1.30)	0.002

All variables are continuous unless otherwise indicated. ^a^Absolute breast FA = unsigned values (|L - R|) of breast volume. ^b^Relative breast fluctuating asymmetry (FA) = 100 × |L - R| breast volume/[|L + R| breast volume] 0.5. ^c^For subgroup of post-menopausal women. When age at menopause is included in the model body mass index (BMI), height, number of pregnancies and parenchyma types P1 and P2 become insignificant, and mean breast volume and parenchyma type DY have borderline significance. Absolute breast FA and relative breast FA remain significant effects. CI, confidence interval.
